# Interactions Between Neurokinin B and Kisspeptin in Mediating Estrogen Feedback in Healthy Women

**DOI:** 10.1210/jc.2016-2132

**Published:** 2016-09-16

**Authors:** Karolina Skorupskaite, Jyothis T. George, Johannes D. Veldhuis, Robert P. Millar, Richard A. Anderson

**Affiliations:** MRC Centre for Reproductive Health (K.S., J.T.G., R.A.A.), The Queen's Medical Research Institute, University of Edinburgh, Edinburgh EH16 4TJ, United Kingdom; Endocrine Research Unit (J.D.V.), Center for Translational Science Activities, Mayo Clinic, Rochester, Minnesota 55905; and Mammal Research Unit and Centre for Neuroendocrinology (R.P.M.), University of Pretoria, 0028 Pretoria, South Africa and MRC Receptor Biology Unit, Institute for Infectious Diseases and Molecular Medicine, University of Cape Town, 7925 Observatory, South Africa

## Abstract

**Context:**

Kisspeptin and neurokinin B (NKB) are obligate for normal gonadotropin secretion, but their hierarchy is unexplored in normal women.

**Objective:**

To investigate the interaction between kisspeptin and NKB on estrogen-regulated LH secretion.

**Design:**

Women were treated with neurokinin-3 receptor (NK3R) antagonist followed by transdermal estradiol to induce LH secretion 48 hours later, with kisspeptin-10 or vehicle infusion during estrogen administration in a 2-way crossover study.

**Setting:**

Clinical research facility.

**Patients or other participants:**

Healthy females with regular menses.

**Intervention(s):**

NK3R antagonist AZD4901 40 mg twice daily orally was taken from cycle day 4–6 for 6 days (n = 10, with 10 no treatment controls). Transdermal estradiol patches (200 μg/d) were applied after 5 days of NK3R antagonist treatment. At 24-hour estradiol treatment, women were randomized to 7-hour kisspeptin-10 (4 μg/kg/h) or vehicle iv infusion, with the alternate infusion in a subsequent cycle.

**Main outcome measure(s):**

Plasma gonadotropin and estradiol secretion.

**Results:**

After an initial suppression, LH secretion was increased 48 hours after estradiol treatment. Kisspeptin-10 increased LH secretion during the inhibitory phase, and LH remained elevated beyond the discontinuation of kisspeptin-10 infusion. NK3R antagonist decreased LH pulse frequency (0.5 ± 0.2 vs 0.7 ± 0.2 pulses/h, *P* < .05) and stimulated FSH response to kisspeptin-10 infusion (10.7 ± 11.0 vs 5.0 ± 3.6 IU/L, *P* < .05) with a nonsignificant rise in LH. The duration of LH response was blunted, with LH being lower at 48 hours (7.5 ± 4.8 vs 15.0 ± 11.4 IU/L, *P* < .05).

**Conclusions:**

These data demonstrate that NKB signaling regulates GnRH/LH secretion in normal women, and is predominantly proximal to kisspeptin in mediating estrogenic positive and negative feedback on LH secretion.

## Abstract (precis)

In a model of the mid-cycle LH surge by administration of estradiol in healthy women,neurokinin B antagonism reduced LH pulse frequency and the duration of kisspeptin-10 stimulated LH secretion

Sex steroid feedback regulates the pulsatile release of hypothalamic GnRH, thereby controlling gonadotropin (LH and FSH) secretion and gonadal function (1). During the early follicular phase of the menstrual cycle, estrogen feedback is inhibitory, but during the late follicular phase, estrogenic feedback stimulates GnRH secretion, culminating in the midcycle LH surge that triggers ovulation. Neuroendocrine mechanisms involved in these pathways and the switch from negative to positive estrogen feedback in the late follicular phase remain unclear.

Kisspeptin and neurokinin B (NKB), neuropeptides partially coexpressed by a population of neurons that also express the opiate, dynorphin, are now recognized as central to the regulation of human reproduction. Patients with loss-of-function mutations in kisspeptin, NKB, or their respective receptors (kisspeptin receptor and neurokinin-3 receptor [NK3R]) show hypogonadotropic failure of pubertal progression (2–5), whereas activating mutations in kisspeptin receptor are associated with precocious puberty (6). Experimental characterization of the relative roles played by kisspeptin and NKB, as well as their functional hierarchy, has been largely carried out in nonhuman models (7–11). In patients with genetic defects inactivating NKB signaling, exogenous kisspeptin, administered using a regimen shown to be maximally stimulatory in healthy volunteers (12), restored LH pulse frequency to normal (13). This, and concordant data from animal models (7, 8), has led to the conclusion that central NKB signaling is functionally upstream of kisspeptin. Data from animal studies of administration of exogenous NKB are discordant, with both stimulatory and inhibitory effects on LH secretion being reported (9–11), whereas it elicited little effect on gonadotropin secretion in a human study (14).

In women, gonadotropin response to exogenous kisspeptin is dependent on the sex-steroid milieu (15) and is greatest in the late follicular phase of the menstrual cycle (16–18), suggesting a role for kisspeptin in the preovulatory positive estrogenic drive to GnRH/LH secretion. Exogenous kisspeptin can increase LH secretion sufficiently to induce oocyte maturation after ovarian stimulation (19), but the role of kisspeptin in physiological positive estrogen feedback is unclear. Involvement of kisspeptin in the ovulatory LH surge in rats and sheep is demonstrated by loss of the LH surge during kisspeptin receptor antagonist treatment (20, 21). The effect of kisspeptin appears largely through an increased frequency of pulsatile GnRH secretion (12, 22, 23), which preferentially stimulates LH over FSH secretion from gonadotrophs (24). Recent data from animal models indicate that administration of an NKB receptor antagonist can slow LH pulsatility (25), and this has also been demonstrated in women with polycystic ovary syndrome (26) where LH pulse frequency is often increased.

We investigated the role of kisspeptin and NKB signaling in the regulation of positive estrogen feedback in women by administration of an NKB receptor antagonist and an infusion of kisspeptin-10 during exogenous estrogen administration. We hypothesized that in this model of estrogen-induced LH secretion, kisspeptin would augment LH secretion and that pharmacological blockade of NKB signaling would reveal the functional hierarchy between kisspeptin and NKB in generating the preovulatory LH surge and in modulating GnRH/LH pulsatility.

## Materials and Methods

### Participants

Twenty healthy women, aged 18–45 years with regular menstrual cycles (25–35 d), were recruited from the community to this study, which was approved by South East Scotland Research Ethics Committee (reference 09/S1101/67); all volunteers provided informed written consent. Subjects were not taking steroidal contraception, had normal physical examination, and full blood count, renal function, electrolytes, liver function, and electrocardiogram were within normal limits.

### Study drugs

Kisspeptin-10 was custom synthesized under Good Manufacturing Practice standards (Bachem GmbH) (12). One milligramof kisspeptin-10 was dissolved in 5-mL sterile normal (0.9%) saline immediately before infusion. The syringe and line for infusion were first coated for 30 minutes with kisspeptin-10 to minimize peptide loss from adherence to the plastic. Sterile normal saline was infused as vehicle. The specific NK3R inhibitor AZD4901, formulated as 20-mg tablets, was gifted by AstraZeneca UK. Transdermal patches releasing 200-μg 17β-estradiol per 24 hours (Janssen) were used as exogenous estradiol treatment (27).

### Protocol

**Figure 1.**
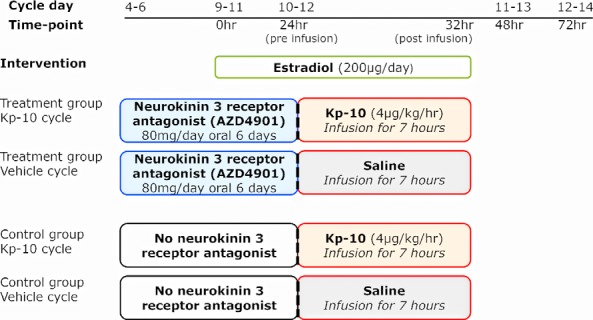
Study protocol diagram. Follicular phase administration of transdermal estradiol was used to induce LH secretion 48 hours later as a model of the midcycle LH surge in women. Ten healthy women were administered NK3R antagonist AZD4901 from cycle day 4–6 for 6 days, matched to 10 women having no treatment. Transdermal estradiol was applied after 5 days; 24 hours later, women were randomized to 7 hour of kisspeptin-10 (Kp-10) or vehicle infusion, returning in a subsequent cycle for the alternate infusion. Reproductive hormones were measured throughout the study and LH pulsatility assessed during 10 minutes of blood sampling for 8 hours.

To standardize estrogen exposure and the onset of increased LH secretion, we used a model of follicular phase administration of transdermal estradiol (200 μg/d), which initially suppresses then at 48-hour increases LH secretion (28). In preliminary studies (Supplemental Figure 1), we confirmed that LH secretion at 48 hours is increased to the same extent if the patches were removed at 32 hours or continued till 72 hours: for the main study, therefore, patches were removed at 32 hours. Sample size was based on previous proof of concept mechanistic studies (12, 15). Twenty women were randomly allocated to NK3R antagonist (AZD4901) 40 mg oral twice daily starting from cycle day 4–6 for 6 days, or no treatment (Figure 1). Two transdermal estradiol patches were administered after 5 days (time 0 h), in the late follicular phase (cycle d 9–11, according to the day of starting AZD4901). At 24 hours of estradiol treatment, volunteers attended our clinical research facility for 8 hours. After an hour of baseline sampling, volunteers were randomized (using sealed envelopes) to receive a continuous iv infusion of kisspeptin-10 (4 μg/kg/h) or vehicle for 7 hours. In the NK3R antagonist treatment group, the last dose of AZD4901 was on the morning of kisspeptin-10 or vehicle administration. Estradiol patches were removed at the end of the infusion, ie, 32 hours after application. Volunteers attended for further measurement of reproductive hormones at 48 and 72 hours. In a subsequent menstrual cycle, all women returned to receive the alternate infusion of kisspeptin-10 or saline. Those receiving NK3R antagonist had at least 1 wash out cycle between treatment cycles. To compare the effect of exogenous vs endogenous estrogen on kisspeptin-10 response, another group of 10 women received iv kisspeptin-10 (4 μg/kg/h) infusion for 7 hours on cycle day 10–12 without exogenous estrogen treatment, with reproductive hormone measurements at equivalent time points.

### Blood sampling and hormone assays

Peripheral venous blood was sampled for LH, FSH, and estradiol in the treatment group on the day of commencing NK3R antagonist and in both control and treatment groups before estradiol treatment (0 h) and then at 24, 32, 48, and 72 hours. During the 8-hour visit, blood samples were collected via an indwelling iv cannula at 10-minute intervals for assessments of LH pulsatility; FSH was measured hourly. Blood samples were centrifuged at 4°C for 10 minutes at 3000 rpm, and serum was frozen at −20°C or below until analysis. LH and FSH were determined by ELISA as previously described (12). 17β-estradiol was measured by ELISA (Demeditec Diagnostics). Inter- and intraassay coefficient of variation for all hormones was less than 5% at the concentrations measured. Lower detection limit for LH and FSH was 0.1 IU/L and for estradiol 20 pmol/L.

### Statistical analysis

ANOVA was used to analyze preliminary data on LH changes with time in the model. For the primary endpoints, hormone concentrations were compared between the 4 treatment groups at specific time points using ANOVA with repeated measures as appropriate. If there was overall significance, post hoc analysis was performed with Bonferroni's correction for multiple comparisons, comparing all 4 treatments simultaneously at each time point. The relationship between the timing of peak LH and treatment was assessed by χ^2^ test. Pearson correlation coefficient was computed to assess the relationship between estradiol concentrations and LH response to kisspeptin-10.

The number of LH pulses, secretory mass of LH per pulse, basal (nonpulsatile), and pulsatile (integral of dual amplitude and frequency regulation) LH secretion were identified by an established deconvolutional algorithm with cluster analysis (93% sensitivity and specificity) (29, 30) blinded to treatment allocation. Approximate entropy (ApEn), a measure of orderliness, was also estimated for the pattern of LH secretion. Deconvolutional estimates and mean hourly hormone changes were not calculated for one woman in each group, as full 8-hour sampling data were not obtained. ANOVA was used to assess changes in LH pulsatility parameters between the 4 groups, with post hoc testing as above.

Data are presented as mean ± SD. Data not normally distributed were log-transformed before statistical analysis, resulting in a distribution that approximated a normal distribution. Differences were regarded as significant at a 2-sided *P* < .05. The statistical software package GraphPad Prism (GraphPad) was used.

## Results

Baseline age, Body Mass Index, and the menstrual cycle length were comparable between the subjects in the control and the treatment group, as were baseline LH, FSH, and estradiol levels in vehicle and kisspeptin-10 cycles within the group (Table 1).

**Table 1.** Baseline Characteristics of Women in the Control and the Treatment Group Undergoing Vehicle and Kisspeptin-10 Infusion

	Control Group		Treatment (NK3Ra) Group		*P* Value
n	10		10		
Age (y)	35 ± 5.8		35 ± 5.4		ns
BMI (kg/m^2^)	25 ± 4.6		28 ± 6.9		ns
Cycle length (d)	29 ± 1.8		28 ± 1.4		ns

	Vehicle Cycle	Kp-10 Cycle	*P* Value	Vehicle Cycle	Kp-10 Cycle	*P* Value
Menstrual cycle day	9.2 ± 0.8	9.4 ± 0.8	ns	4.4 ± 0.5	4.8 ± 0.8	ns
LH (IU/L)	4.8 ± 2.1	5.6 ± 2.0	ns	5.0 ± 1.8	4.9 ± 1.8	ns
FSH (IU/L)	4.3 ± 1.2	5.3 ± 2.6	ns	6.2 ± 1.8	5.5 ± 1.3	ns
Estradiol (pmol/L)	274 ± 126	287 ± 157	ns	121 ± 54	154 ± 47	ns

Data are shown as mean + SD; BMI, Body Mass Index; Kp-10, Kisspeptin-10; ns, not significant. Note that baseline data on control and treatment groups in lower part of the table reflect sampling at different stages of the menstrual cycle.

### Model validation for estrogen-induced LH secretion

Treatment with exogenous estrogen for 32 hours increased serum estradiol concentrations as expected (*P* < .0001) (Supplemental Figure 1). Serum LH was initially suppressed at 32 hours of estrogen treatment, then increased at 48 hours, which persisted at 72 hours (all *P* < .05 vs 0 h). FSH concentrations were significantly lower at 24 (*P* < .01) and 32 hours (*P* < .0001) but were not higher at 48 and 72 hours compared with baseline. This confirms that with this regimen, estrogenic negative feedback is followed by increased LH secretion, thus standardizing estrogen exposure and the time course of changes in LH secretion.

### Kisspeptin-10 stimulates gonadotropin secretion

**Figure 2.**
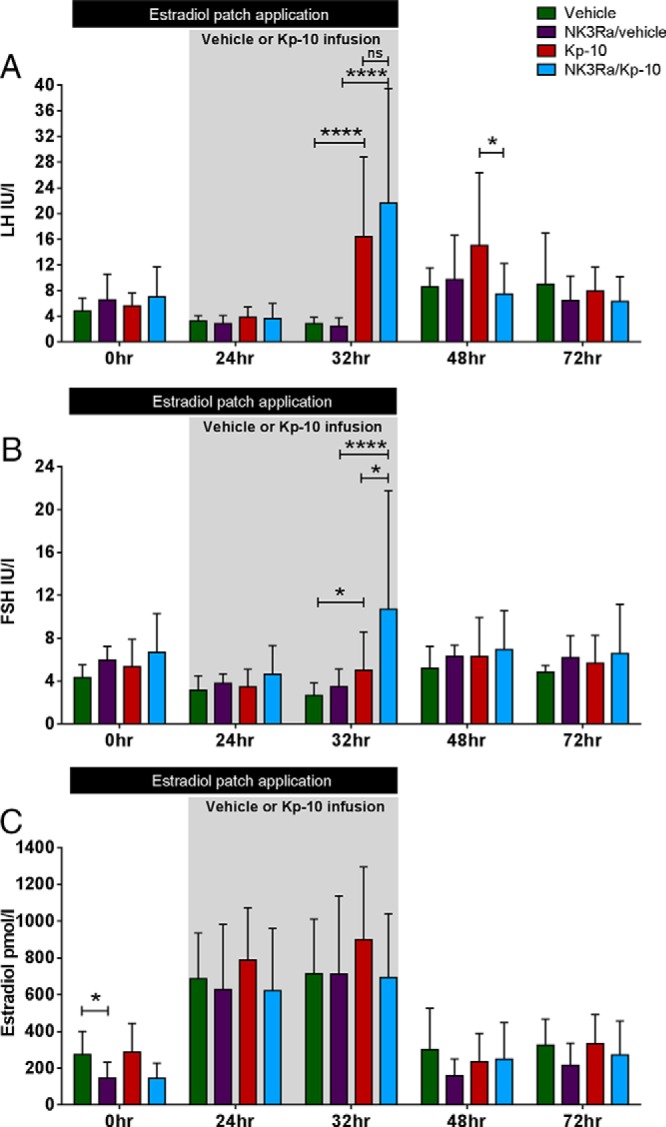
Comparison of mean LH (A), FSH (B), and estradiol (C) response to an infusion of kisspeptin-10 (Kp-10) and vehicle in 10 control and 10 NK3R antagonist-treated women in the model of estrogen-induced LH secretion. Two estradiol patches releasing a total of 200-μg estradiol/d were applied between 0 and 32 hours. The infusion period of kisspeptin-10 and vehicle was between 24 and 32 hours. Data presented as mean ± SD. *, *P* < .05; ****, *P* < .0001.

**Figure 3.**
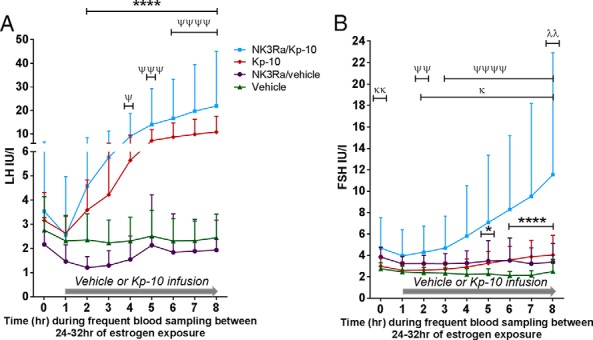
LH (A) and FSH (B) levels an hour before and during 7 hours of vehicle or kisspeptin-10 (Kp-10) infusion in the control group (n = 9) and in the treatment (NK3R antagonist) group (n = 9). The infusion period of kisspeptin-10 and vehicle was between 24 and 32 hours of estrogen administration. Data presented as mean ± SD. Statistical analysis by two-way ANOVA determined statistically lower LH levels between vehicle and NK3Ra-treated women (*P* < .0001), although Bonferroni's post hoc multiple comparison test found no significant changes at specific time points. For vehicle vs kisspeptin-10 infused controls: *, *P* < .05; ****, *P* < .0001. For kisspeptin-10 infusion in controls vs NK3Ra: λλ, *P* < .01. For vehicle vs NK3Ra: κ, *P* < .05; κκ, *P* < .01. For vehicle vs kisspeptin-10 in NK3Ra-treated women: Ψ, *P* < .05; ΨΨ, *P* < .01; ΨΨΨ, *P* < .001; ΨΨΨΨ, *P* < .0001.

During estrogen administration, kisspeptin-10 stimulated LH secretion to 16.4 ± 12.4 IU/L at the end of infusion vs 2.9 ± 1.0 IU/L after vehicle administration (*P* < .0001) (Figure 2A). The time course of this response is shown in Figure 3A. Kisspeptin-10 induced LH secretion persisted beyond the discontinuation of the infusion with higher peak LH compared with controls at 48 hours (9.3 ± 1.9 vs 21.6 ± 13.0 IU/L, *P* = .007) (Supplemental Table 1). Clarification of the impact of exogenous estradiol on this response was demonstrated in a separate group of women receiving kisspeptin-10 infusion only in the late follicular phase without exogenous estrogen administration, who showed a similar acute increase in LH secretion correlating positively with estradiol concentration (*r*^2^ = 0.63, *P* = .006), but of a shorter duration (48 h: 6.8 ± 5.8 IU/L vs 15.0 ± 11.4 with estrogen treatment, *P* < .01) (Supplemental Figure 2). All subjects in the endogenous estrogen group had peak LH at the end of kisspeptin-10 infusion, whereas in exogenous estrogen-treated subjects, the kisspeptin-10-induced peak LH persisted beyond kisspeptin-10 infusion with 50% of women having peak LH at 32 hours and 50% at 48 hours (*P* < .01, Supplemental Table 1).

FSH secretion was also significantly higher at the end of kisspeptin-10 infusion compared with vehicle in the control group (*P* < .05) but not different to baseline (0 h) (Figures 2B and 3B). As expected with this model of exogenous estradiol administration, estradiol concentrations were similar in kisspeptin-10 and vehicle-infused controls (Figure 2C).

### NK3R antagonist has differential effects on LH and FSH secretion

Serum LH levels did not change after 5 days of NK3R antagonist treatment (before the estradiol patches were applied) when compared with either pretreatment concentrations (pre-NK3Ra 5.0 ± 1.8 vs 5 d NK3Ra 6.6 ± 4.0 IU/L, nonsignificant [ns]) or to controls (Figure 2A). Overall, there was no difference in LH concentrations and the timing of peak LH in controls vs NK3Ra-treated women (Supplemental Table 1 and Figure 2A). To detect subtle changes in hormone secretion potentially overlooked by single time point blood sampling, analysis of hourly LH for 8 hours after dose showed that overall LH secretion was lower in NK3Ra-treated women compared with controls (*P* < .0001) (Figure 3A), although post hoc analysis indicated no significant differences in LH levels at any individual hourly time point.

FSH concentrations appeared higher throughout treatment with NK3R antagonist compared with controls (Figure 2B) and were significantly higher in NK3Ra-treated women throughout the 8-hour period (ie, during saline infusion, *P* < .0001) (Figure 3B). This may reflect that serum estradiol concentrations were significantly lower after 5 days of treatment with NK3R antagonist compared with controls (*P* < .05) (Figure 2C) and comparable with estradiol levels before NK3R antagonist administration (pre-NK3Ra, 121 ± 54 vs 145 ± 87 pmol/L; after 5 d of NK3Ra, ns).

### Effect of NK3R antagonist on the gonadotropin response to kisspeptin-10

NK3R antagonist nonsignificantly increased kisspeptin-10 stimulated LH secretion at 32 hours (21.6 ± 17.8 with NKB antagonist vs 16.4 ± 12.4 IU/L kisspeptin-10 alone, *P* = .41) (Figures 2A and 3A). The FSH response to kisspeptin-10 was, however, significantly more pronounced in the presence of NK3Ra (10.7 ± 11.0 vs 5.0 ± 3.6 IU/L at 32 h; *P* < .05) (Figure 2B) and throughout the 7-hour infusion (*P* < .0001) (Figure 3B).

However, NK3Ra blunted the duration of kisspeptin-10-induced LH secretion, with significantly lower LH at 48 hours (15.0 ± 11.4 vs 7.5 ± 4.8 IU/L, *P* < .05) when compared with kisspeptin-10 infused controls, whereas FSH showed no significant difference (Figure 2). There were related changes in the timing of the LH peak (although not statistically significant), which was at 32 hours in 9/10 NK3Ra-treated women in response to kisspeptin-10 infusion, compared with kisspeptin-10 treated controls whose LH peak timing was evenly divided between 32 and 48 hours (Supplemental Table 1).

### NK3R antagonist impedes estradiol-dependent kisspeptin-10 response

**Figure 4.**
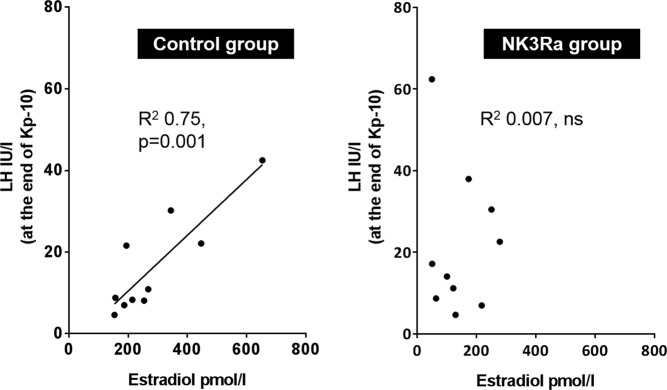
Correlation between endogenous estradiol and LH response to kisspeptin-10 in controls (left) and NK3R antagonist-treated (right) women. Kisspeptin-10 response on LH secretion is positively related to endogenous estradiol levels, whereas this correlation is not seen during NK3R antagonist treatment. Analysis of residuals demonstrated normal distribution.

The relationship between LH response to kisspeptin-10 and estradiol exposure, and the influence thereon of NK3Ra treatment, was investigated by analyzing LH concentration at the end of kisspeptin-10 infusion in relation to endogenous estradiol concentrations at 0 hours (ie, before transdermal estradiol application). There was a strong positive correlation in controls (*r*^2^ = 0.75, *P* = .001) (Figure 4). However, in NK3Ra-treated women, the LH response to kisspeptin-10 showed no such relationship (*r*^2^ = 0.007, ns). Very similar results were obtained when the analysis was based on estradiol concentrations after 24 hours of patch administration (*r*^2^ = 0.65, *P* = .005 in controls; *r*^2^ = 0.03, ns in NK3Ra-treated women).

### Interaction between NK3R antagonist and kisspeptin-10 in regulating LH pulsatility

**Figure 5.**
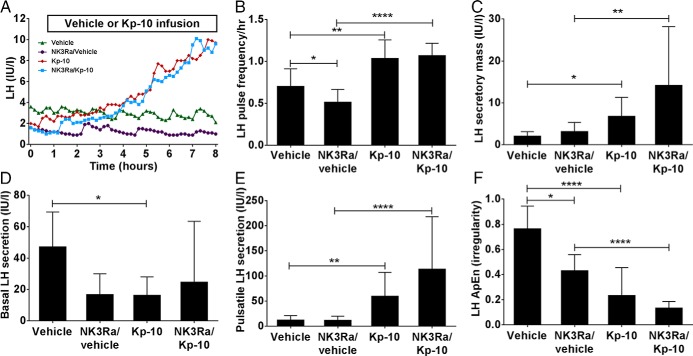
Analysis of 8 hour LH secretory pattern during vehicle and kisspeptin-10 (Kp-10) in controls and NK3Ra-treated women. A, Illustrative LH pulse profile from one subject undergoing vehicle (green triangles), NK3R antagonist (purple circles), kisspeptin-10 (red diamonds), and NK3R antagonist followed by kisspeptin-10 (blue squares) treatment visits. Mean LH pulse frequency (B), secretory mass of LH per pulse (C), basal (nonpulsatile) LH secretion (D), pulsatile LH secretion (E), and the relative orderliness/regularity of LH secretory pattern (F) during vehicle and kisspeptin-10 infusion with (n = 9) and without (n = 9) pretreatment with NK3R antagonist. Mean ± SD. *, *P* < .05; **, *P* < .01; ****, *P* < .0001.

LH pulse frequency increased from 0.7 ± 0.2 pulses/h in vehicle cycle to 1.0 ± 0.2 pulses/h during kisspeptin-10 infusion (*P* < .01) (Figure 5, A and B). NK3R antagonist reduced LH pulsatility to 0.5 ± 0.2 pulses/h (*P* < .05 vs vehicle-infused controls), but administration of kisspeptin-10 to NK3Ra-treated women restored LH pulse frequency to that observed in kisspeptin-10-infused controls. Thus, although NK3Ra slowed LH pulsatility in estrogen-treated women, it did not affect the response to kisspeptin-10, indicating that kisspeptin effects are downstream of NKB signaling.

Secretory mass of LH per pulse was increased similarly during infusion of kisspeptin-10 compared with vehicle in both control (*P* < .05) and NK3R antagonist-treated women (*P* < .01) (Figure 5C). NK3Ra antagonist did not reduce LH secretory mass per pulse.

Consistent with increased LH pulse frequency, basal LH secretion decreased and pulsatile LH secretion increased during kisspeptin-10 infusion in the control group (*P* < .05 vs vehicle) (Figure 5, D and E). Basal LH secretion appeared lower in NK3Ra-treated women when compared with controls, but there was no effect on pulsatile LH secretion. Kisspeptin-10 induced the same changes in NK3Ra-treated women as in controls, with no change in basal and an increase in pulsatile LH secretion (*P* < .0001).

The regularity of LH secretory pattern was assessed by ApEn. Both kisspeptin-10 infusion and NK3Ra separately imposed greater orderliness (lower ApEn) in LH secretion (*P* < .05) (Figure 5F). This was increased further in NK3Ra-treated women during kisspeptin-10 infusion (*P* < .0001 vs NK3Ra alone) (Figure 5F).

## Discussion

In a model of LH modulation by estrogen administration in women, exogenous kisspeptin-10 stimulated LH secretion, the extent of which reflected estradiol concentrations. Pharmacological blockage of NKB-NK3R signaling slowed LH pulsatility and shortened the duration of kisspeptin-mediated LH secretion with “sharpening” of the LH response, and strikingly abolished the relationship between estradiol and LH response to kisspeptin. Taken together, these data support a central role for kisspeptin in the modulation of GnRH/LH secretion, and although NKB signaling is largely upstream of kisspeptin as previously reported (13), both pathways interact in determining the timing and characteristics of estrogenic negative and positive feedback on LH secretion.

The stimulatory effect of exogenous kisspeptin on LH secretion in women is dependent on the sex steroid environment (15–18). This response is initially limited but increases markedly in the late follicular phase of the menstrual cycle when estradiol levels are rising (16–18). For most of the cycle, GnRH and thus LH secretion are inhibited by negative feedback from estradiol (and progesterone in the luteal phase), thus the low responsiveness to kisspeptin administration in previous studies is consistent with endogenous kisspeptin signaling being suppressed by the inhibitory steroidal actions, presumably at the GnRH neuron and/or gonadotroph level. Conversely, the enhanced LH response in the later follicular phase may indicate the development of increased endogenous kisspeptin signaling in the lead up to the midcycle surge. This is supported by animal studies, where kisspeptin expression is highest after an estrogen challenge in the anteroventral periventricular nucleus (the site of positive estrogen feedback in rodents) in ovariectomized mice (31) and at the time of GnRH/LH surge in sheep (32), but is prevented by administration of kisspeptin receptor antagonist (20, 21). The present data suggests a role of estradiol in modulating LH response to kisspeptin-10 infusion, which persisted well beyond the pharmacokinetic clearance of the exogenous kisspeptin-10. The striking positive relationship between estradiol concentration in the late follicular phase and the LH response to kisspeptin-10 infusion lends further support for the involvement of kisspeptin in estrogen feedback, as recently demonstrated for kisspeptin-54 (33).

The present data demonstrate that NK3R antagonist treatment, in an environment of high estrogenic negative feedback, reduced LH secretion and pulse frequency, whereas in the presence of kisspeptin-10 had a stimulatory effect on the secretion of both gonadotropins, but with a shorter duration of LH response. Hitherto, NK3R antagonists have been demonstrated to suppress LH secretion in states of high LH output, such as in women with Polycystic Ovary Syndrome (26), or in the ovariectomized ewe and castrate monkeys (25, 34), and in intact female monkeys a delay of surge like, but no decrease in basal LH secretion was observed (25). Although it appears that the suppression of LH secretion by the present dose and regimen lasted only a few hours, this was sufficient to significantly lower estradiol concentrations after 5 days of NK3R antagonist treatment (ie, before the estrogenic treatment) compared with controls. Although this demonstrates a role of NKB in the regulation of LH secretion, NK3R antagonist had no effect on the timing of peak LH secretion in this model of estrogen administration, which is consistent with a lack of effect of NK3R antagonist on the estrogen induced LH surge seen in ovariectomized ewes (34). The mechanisms critical for progression to positive estrogen feedback therefore appear to be largely independent of NKB but dependent on kisspeptin, consistent with rodent neuroanatomical data (31, 35). A recent study using a different NK3R antagonist in normal women also showed a temporary suppression of LH levels lasting few hours after dosing, but no overall decrease in basal LH secretion after treatment throughout the follicular phase (34). NK3Ra did, however, delay LH surge in some women, probably as a consequence of delayed preovulatory estradiol rise, but the study did not assess the effect of NKB antagonism at the time of the switch from negative to positive estrogen feedback, when NKB might be no longer critical (36). Unlike in the present study, no effect of NK3Ra on FSH secretion was observed (35).

We have previously demonstrated that infusion of kisspeptin-10 can restore LH pulsatile secretion in men and women with inactivating mutations in NKB signaling, indicating that kisspeptin is functionally downstream of NKB in LH pulse generation (13). This is supported by the inability of the NK3R agonist senktide to stimulate LH secretion in *Kiss1r* knockout mice (7). Consistent with this overall hierarchy, NKB antagonism (active during kisspeptin-10 administration as half-life of AZD4901 is 8.5 h) (37) did not prevent the stimulatory effect of kisspeptin-10 infusion on LH secretion. NKB antagonist, however, shortened the LH response to kisspeptin-10, affecting its timing by reducing the variability of peak LH secretion, and disrupted the relationship between LH response and estradiol concentrations. These findings suggest a more complex interaction than a linear pathway between those neuropeptides at the time of the midcycle LH surge, but are also consistent with NK3R antagonist reducing endogenous kisspeptin stimulation of GnRH as a contribution to the observed effect.

LH pulse frequency increases in the late follicular phase, culminating in the midcycle LH surge (38). Exogenous kisspeptin stimulates pulsatile LH secretion (12, 13, 22, 23, 39), but its role as a potential contributor to positive estrogen feedback has not been previously investigated. In this study, the increase in LH secretion during kisspeptin-10 infusion included increased LH pulse frequency and mass-per-secretory pulse. This resulted in a larger proportion of total LH secretion occurring in pulsatile bursts, and the regularity of LH secretory pattern showed greater orderliness in the lead up to the stimulatory phase of response to estrogen. Deconvolution analysis also indicated changes in the nature of the pulsatile LH secretion after NK3R antagonist administration, with reduced basal secretion and ApEn, indicating a more orderly, slowed pattern of LH and by inference GnRH secretion. The increase in LH pulse frequency resulting from kisspeptin-10 infusion and the slowing in LH pulsatility with NK3R antagonist administration both increased the regularity and orderliness of LH secretion and may be the basis for the reduced variability in the timing of peak LH secretion as well as shortened duration of stimulated LH secretion in response to kisspeptin-10. Consistent with some aspects of our findings, estrogen-induced LH surges were preserved in ovariectomized NK3R antagonist-treated ewes, although the onset-to-peak time was delayed (34). In sheep, the NK3R agonist senktide increased LH secretion, resembling “surge-like” LH levels (9), whereas in monkeys, NK3R antagonist abolished LH surge, ovulation, and subsequent progesterone rise (25). Although our data primarily indicate that NKB signaling is largely upstream of kisspeptin signaling in mediating estrogenic positive effects, it clearly has a modulatory role in determining the pattern and duration of GnRH secretion during estrogen positive feedback.

A stimulatory effect of kisspeptin alone on FSH has been minimal and inconsistent in previous studies (16–18, 40) but was robustly demonstrated in this model and was not prevented by NK3R antagonist treatment. NK3R antagonist also increased FSH secretion and markedly augmented stimulation by kisspeptin-10. These findings are consistent with well-established data from animal models showing that high GnRH pulse frequency favors LH secretion, whereas low pulse frequency favors FSH secretion (24), and that this is the main drive to follicular estrogen production, the reduction in both of which (and presumed reduced inhibin production) is likely to have resulted in the observed increased FSH secretion. The differential effects of NK3Ra on FSH vs LH response to kisspeptin-10 are also similar to the effects in patients with NKB defects (13).

Although the present study has clear strengths (the use of specific NK3R antagonist, detailed LH pulse profiling and blinded pulse analysis), there are weaknesses. The sample size is small, and placebo was not administered to the control group receiving no NK3Ra. The limited LH suppression by the NK3R antagonist might be due to the small sample size and the dose of AZD4901 may be low compared with those used in animal studies, limiting the response (25). Statistical analyses included adjustment for α for multiplicity of comparisons, but studies such as these should be regarded as mechanistic explorations. Moreover, this model of LH secretion after exogenous estrogen administration may not fully replicate physiological positive estrogen feedback in the preovulatory state.

In summary, using estrogen to standardize LH secretion in women to model the midcycle LH surge, we have shown that the increase in LH secretion by kisspeptin-10 infusion is related to estradiol exposure. We show for the first time that NK3R antagonist reduced LH pulsatility in healthy women. Assessment of the interaction between kisspeptin and NKB showed that the duration of kisspeptin-mediated LH secretion was shortened by the NK3R antagonist, and the quantitative relationship with estradiol exposure abolished. These data thus indicate that NKB pathways regulate GnRH/LH secretion in women, are predominantly upstream of kisspeptin signaling in mediating estrogen feedback, but modify this kisspeptin response. This extends our understanding of these critical events in human reproduction.
